# Supplementary material for: The Gender Impact Assessment among Healthcare Workers in the SARS-CoV-2 Vaccination—An Analysis of Serological Response and Side Effects
Source: Vaccines (Basel). 2021 May 18;9(5):522. doi: 10.3390/vaccines9050522 (PMC8158485; doi:10.3390/vaccines9050522)
Supplement: Supplementary file 1 [file vaccines-09-00522-s001.zip › vaccines-1206999-supplementary.pdf]

**Table S1: The distribution in terms of sex (64% females; 36% males) and age of the all the healthcare workers (HW) at IRCCS San Raffaele Hospital.**

| | age group (y) | n |
|---|---|---|
| Females | <48 | 2473 |
| | 48-52 | 584 |
| | >52 | 1049 |
| **Total** | | **4106** |
| Males | <40 | 1002 |
| | 40-60 | 1015 |
| | >60 | 272 |
| **Total** | | **2289** |
| **Total HW in OSR** | | **6395** |

**Table S2: Serological tests in the population with the natural immunity to SARS-CoV-2**. Median values and IQR (Q1 and Q3) are reported, disaggregated by sex (M: male; F: female) and age groups.

| **Age *group*** | ***M*** | | | ***F*** | | | **Age *group*** |
|---|---|---|---|---|---|---|---|
| | **Q1** | **Median** | **Q3** | **Q1** | **Median** | **Q3** | |
| *<40 y* | 7 | 27 | 65 | 8 | 27 | 69 | *<48 y* |
| *41-60 y* | 10 | 38 | 83 | 15 | 42 | 124 | *48-52 y* |
| *>60 y* | 24 | 59 | 105 | 10 | 43 | 130 | *>52 y* |

**Table S3: Serological values at T1 of the reactive subjects to the first vaccine dose.** Median values and IQR (Q1 and Q3) are reported. In males, the statistically significant difference is present between <40 y and 40-60y, between 40-60y and >60y, and between <40y and >60y (p<0.0001). In females, among the different groups the following p values are reported: <48y vs >52y, p<0.0001; <48y vs 48-52y, p=0.002; 48-52y vs >52y, p=0.002.

| **Age *group*** | ***M*** | | | ***F*** | | | **Age *group*** |
|---|---|---|---|---|---|---|---|
| | **Q1** | **Median** | **Q3** | **Q1** | **Median** | **Q3** | |

| | | | | | | | |
|---|---|---|---|---|---|---|---|
| *<40 y* | 27,1 | 62,9 | 145 | 24.4 | 54.1 | 129,5 | *<48 y* |
| *41-60 y* | 10,3 | 29,6 | 84,9 | 19 | 45 | 90 | *48-52 y* |
| *>60 y* | 4,5 | 12,1 | 35,7 | 9 | 29 | 81 | *>52 y* |

**Table S4: Serological values at T2 to evaluate the response to the COVID-19 vaccine.** Median values and IQR (Q1 and Q3) are reported, disaggregated by sex and age groups.

| **Age *group*** | ***M*** | | | ***F*** | | | **Age *group*** |
|---|---|---|---|---|---|---|---|
| | **Q1** | **Median** | **Q3** | **Q1** | **Median** | **Q3** | |
| *<40 y* | 1185,5 | 1844 | 2500 | 1179,2 | 2054,5 | 2500 | *<48 y* |
| *41-60 y* | 693,2 | 1495 | 2264,5 | 1053,5 | 1609 | 2500 | *48-52 y* |
| *>60 y* | 623,2 | 1235,5 | 1850 | 700 | 1521 | 2500 | *>52 y* |

**Table S5: Percentage of respondents based on disaggregated data by sex and age groups.**

| | **age group (y)** | **%** |
|---|---|---|
| **Respondent females** | <48 | 61 |
| | 48-52 | 15 |
| | >52 | 24 |
| **Respondent males** | <40 | 44% |
| | 40-60 | 43% |
| | > 60 | 13% |
